# Design and Application of a Sawdust–Alginate Biocomposite for Sustainable Cationic Dyes Removal from Aqueous Solutions

**DOI:** 10.3390/polym18091136

**Published:** 2026-05-05

**Authors:** Narcis-Teodor Niță, Elena-Mirela Suceveanu, Florin Marian Nedeff, Lidia Favier, Eugen Herghelegiu, Lăcrămioara Rusu

**Affiliations:** 1 Doctoral Studies School, “Vasile Alecsandri” University of Bacau, 157 Calea Mărășești, 600115 Bacău, Romania; narcisteodor170@gmail.com; 2 Faculty of Engineering, “Vasile Alecsandri” University of Bacau, 157 Calea Mărășești, 600115 Bacău, Romania; florin_nedeff@ub.ro; 3 Ecole Nationale Supérieure de Chimie de Rennes, University of Rennes, CNRS, UMR 6226, CEDEX 7, 35708 Rennes, France; lidia.favier@ensc-rennes.fr

**Keywords:** biosorption, sawdust, natural polymers, malachite green, kinetic models, equilibrium isotherms, wastewater treatment

## Abstract

This study investigates a novel biocomposite material developed by immobilizing sawdust within a calcium alginate matrix (SDA 5%) for the removal of dyes from aqueous solutions. The material was synthesized and comprehensively characterized using FTIR, SEM, and EDS analyses and the pH_pzc_ drift method. Laboratory-scale experiments were performed to evaluate its performance in removing Malachite Green (MG) under varying operational conditions, including initial dye concentration (10–50 mg/L), pH (3–6), and biosorbent dosage (1–6 g/L). At pH 6 and a biosorbent dose of 3 g/L, under constant agitation (130 rpm), SDA 5% achieved removal efficiencies exceeding 95% across all tested MG concentrations. Furthermore, the biosorption capacity increased with increasing initial dye concentration, reaching a maximum value of 15.93 mg/g at an initial MG concentration of 50 mg/L. Nonlinear kinetic modelling revealed that the pseudo-second-order model best described the biosorption process, while equilibrium analysis showed that the Hill and Sips nonlinear isotherm models, followed by Temkin, provided the most accurate fit to the experimental data. These results demonstrate the high biosorption capacity and favorable interaction between MG molecules and the biocomposite surface. Overall, the study highlights sawdust-alginate biocomposites as sustainable, low-cost, and environmentally friendly biosorbents with significant potential for practical wastewater treatment applications.

## 1. Introduction

Wastewater generated by industries such as textiles, leather tanning, paper production, food processing, and hair-dye manufacturing is frequently contaminated with synthetic dyes [1]. Global dye production reaches approximately 8 × 10^5^ tons per year [2], and during manufacturing, about 12% of these dyes are lost, while nearly 20% of the remaining color is discharged into the environment through industrial effluents [3]. The textile industry, in particular, uses large volumes of water, around 100 L per kilogram of fabric, and its dyeing processes generate intensely colored wastewater containing unreacted or excess dyes [4]. These effluents often include a wide range of dye classes, such as cationic, acid, direct, disperse, reactive, and sulfur dyes, many of which exhibit low fixation rates and poor biodegradability, contributing to high pollutant loads [4]. Synthetic dyes released into aquatic systems represent a major ecological concern: they persist due to their complex chemical structures and stability toward physicochemical and microbial degradation, alter the physicochemical properties of surface waters, and can exert toxic, mutagenic, or carcinogenic effects even at low concentrations [5]. When discharged untreated or only partially treated, these dye-contaminated wastewaters negatively impact environmental compartments and pose risks to human health [2].

Among synthetic dye pollutants, cationic dyes are of particular concern due to their extensive industrial use and well-documented environmental and health risks. These dyes persist in the environment because of their complex molecular structures and resistance to biodegradation, leading to accumulation in aquatic systems where they cause toxicity in aquatic organisms, disrupt ecosystems, and inhibit plant growth by reducing light penetration. In humans, exposure to cationic dyes may result in skin irritation, allergic reactions, respiratory problems, and increased carcinogenic risk. A relevant example is Malachite green (MG), a water-soluble triphenylmethane dye widely employed in the textile, food, medical, and paper industries, and commonly used in aquaculture as a parasiticide against helminths, fungi, and protozoan infections [3,6,7]. Numerous studies have demonstrated the severe toxicological effects of MG: it reduces water transparency and disrupts photosynthesis in aquatic plants; induces biochemical alterations in fish; and accumulates in tissues such as the liver, kidneys, muscles, and serum in both its oxidized form (MG) and reduced form (leuco-malachite green, LMG). In mammals, MG exhibits teratogenic and mutagenic properties and has been associated with liver, kidney, spleen, and heart damage, pulmonary toxicity, and carcinogenesis, with its toxicity increasing with exposure duration, concentration, and temperature [8,9]. Despite these known hazards, MG remains in use across several industries. Its removal from aquatic environments is particularly challenging due to its high resistance to light and oxidizing agents, rendering conventional biological and chemical treatments largely ineffective [7]. Consequently, substantial research has focused on developing both conventional and advanced techniques for eliminating residual MG from wastewater [10].

Recently, a wide range of technologies has been explored for the removal of dyes in general, and malachite green (MG) in particular, from wastewater, including advanced oxidation processes (AOPs), electrocoagulation, coagulation–flocculation, membrane filtration, biological treatment, and adsorption, applied either individually or in combination [11,12]. Physicochemical and biological methods such as membrane filtration, ion exchange, photocatalysis, aerobic and anaerobic treatment, electrodialysis, Fenton oxidation, reverse osmosis, ozonation, and adsorption have shown promising results; however, their industrial application remains restricted by high operational costs, energy demands, lengthy treatment times, and substantial production of secondary sludge [11].

When considering AOP-based treatments, several strengths and limitations can be highlighted. Catalytic AOPs involve moderate costs and generate minimal secondary pollution, but they rely heavily on light energy. Fenton-type processes exhibit strong oxidative capacity and rapid reaction rates; however, they require acidic conditions and produce iron-containing sludge. Non-thermal plasma systems have high energy consumption and require specialized equipment, while sulfate-radical AOPs maintain high efficiency over a wide pH range but need activation steps and generate sodium-sulfate by-products. Despite their high degradation efficiency, AOPs can produce toxic intermediates and require substantial energy input, raising concerns about their long-term sustainability [10].

Biological approaches have also been investigated, using bacteria such as *Ochrobactrum* sp. [13], *Pseudomonas* sp. YB2 [14], *Pseudomonas plecoglossicida* MG2 [15], *Pseudomonas veronii* [16], yeasts such as *Rhodotorula mucilaginosa* [17], fungi such as *Aspergillus flavus* [18], and microalgae including *Desmodesmus* sp. [19], *Cosmarium* sp. [20], *Chlorella vulgaris* and *Scenedesmus* sp. [21,22]. Although biodegradation is environmentally friendly and generally non-toxic, its practical scalability remains limited due to high feasibility-study costs, slow degradation rates, restricted efficiency for high MG concentrations, and the dependence on biodegradable pollutants. Furthermore, MG’s chemical stability and biocidal properties considerably hinder microbial degradation, making biological treatment alone insufficient for effective large-scale removal [17,22].

Among the available techniques for dye removal, each method presents specific advantages and limitations, with the optimal choice depending on factors such as cost, efficiency, and environmental impact. However, adsorption has emerged as one of the most promising strategies, as it offers low operational cost, simplicity, minimal energy consumption, high effectiveness even in the presence of toxic contaminants, and limited generation of harmful by-products, particularly when efficient, inexpensive, non-toxic, and readily available biosorbents are employed [23,24,25]. Its performance depends primarily on the chemical composition of the adsorbent surface, the presence of reactive functional groups, and its specific surface area [11]. Conventional adsorbents such as silica gel and activated carbon exhibit high removal capacities, yet their large-scale use is often constrained by high production costs and challenges associated with regeneration. Consequently, increasing research efforts have focused on developing alternative, cost-effective, and environmentally sustainable adsorbents with comparable efficiency [11]. For example, hydrogels based on natural polysaccharides such as cellulose and alginate have gained increasing attention in wastewater treatment due to their porous three-dimensional structure, abundant hydrophilic functional groups, and high adsorption capacity for various pollutants, including dyes and heavy metals [26]. Over the past decades, adsorption-based approaches for MG removal have gained significant attention, leading to the development of numerous new adsorbent materials that have demonstrated highly promising results [10]. Examples include activated carbon derived from *Rumex abyssinicus* [27], *Curcuma caesia*—based activated carbon [28], and tetraethylenepentamine—functionalized activated carbon synthesized via microwave-assisted methods [29], all of which have shown strong adsorption performance toward MG.

The principles of the circular economy, increasingly promoted at the global level, have stimulated the valorization of residual materials generated across diverse industrial sectors [5]. Within this framework, biomass wastes, such as agricultural residues, natural materials, and food or household waste, represent renewable and underexploited resources that can be transformed into high-value materials suitable for adsorption applications. Numerous plant and animal-derived residues, including stems, sawdust, nutshells, bark, eggshells, feathers, leaves, and related by-products, have been successfully converted into adsorbents for the removal of persistent contaminants from wastewater [11]. These waste streams typically possess minimal commercial value and often pose disposal challenges. Therefore, their utilization as adsorbents provides both an economically beneficial and environmentally waste-management strategy [11].

Among biomass-derived resources, woody biomass is particularly noteworthy. The wood-processing industry generates substantial quantities of lignocellulosic residues with significant potential for valorization in applications such as construction materials, enzymatically derived biofuels, cellulose manufacturing, furniture and packaging production, animal bedding, and thermal and electrical energy generation [30]. Lignocellulose biomass comprises cellulose, hemicellulose, and lignin, together with extractive compounds including paraffins, resins, tannins, fats, pigments, and inorganic salts [31]. Despite its abundance and versatility, a considerable portion of this resource remains underutilized, contributing to environmental burdens.

A representative example of lignocellulosic biomass is sawdust, a major by-product of sawing, planing, and cutting operations, generated in large quantities worldwide. Due to its low commercial value, sawdust is often disposed of through uncontrolled practices such as open dumping or incineration, which may result in environmental pollution and toxic emissions [32].

In response to growing environmental concerns, recent studies have highlighted sawdust as a versatile and sustainable material, suitable for applications including fuel production, thermal insulation, construction materials [33,34], composting [35], and particularly as a low-cost adsorbent for wastewater treatment, enabling the removal of dyes, heavy metals, and pharmaceuticals [36].

Its high availability (over 24.25 million m^3^ globally), low cost, renewable origin, and favorable physicochemical characteristics—being dependent on wood species and particle-size distribution—make sawdust an attractive precursor for the development of alternative adsorbents [32].

Effective removal of cationic dyes using sawdust has been widely documented. For example, Abyar et al. (2025) [37] reported a 96.86% removal efficiency for methylene blue, while several studies demonstrated its suitability for MG adsorption owing to the presence of functional groups such as hydroxyl, amine, and carboxyl moieties [38]. Reported maximum MG adsorption capacities include 4.35 mg/g for neem sawdust [39], 62.71 mg/g for rattan sawdust [1], and 83.21 mg/g for beech sawdust at 20 °C [40]. Chemical modification strategies have been shown to markedly enhance adsorption performance. For instance, Wang et al. (2014) [41] reported that *Cinnamomum camphora* sawdust treated with oxalic, citric, and tartaric acids achieved maximum adsorption capacities of 280.3, 222.8, and 157.5 mg/g, respectively. Similarly, Song et al. (2015) [6] demonstrated that triethylamine-modified cypress sawdust reached an adsorption capacity of 697.8 mg/g, representing a substantial improvement over the 94.0 mg/g obtained for the unmodified material.

In parallel, alginate-based adsorbents have also shown excellent potential for MG removal, with reported removal efficiencies of 84.47% [42] and 98.5% [43], and adsorption capacities of up to 628.93 mg/g [44] and 322.58 mg/g [45], underscoring the relevance of biopolymeric materials in sustainable wastewater treatment.

Based on the considerations outlined above, it becomes evident that, in response to increasing environmental concerns, recent research has investigated the potential of sawdust as a low-cost and sustainable adsorbent for wastewater treatment. Its application in the removal of cationic dyes from aqueous solutions has yielded promising results. Converting sawdust into an adsorbent, owing to its high availability, low economic value, and favorable surface properties, not only enhances the efficiency of dye-removal processes but also aligns with the principles of waste valorization and the circular economy by transforming industrial residues into functional materials. To the best of our knowledge, the existing scientific literature does not report the synthesis and use of a biomaterial consisting of sawdust immobilized within a calcium alginate matrix for the biosorption of organic dye pollutants. The novelty of the present work lies in the immobilization of sawdust within a calcium alginate matrix, which combines the adsorption functionality of lignocellulosic biomass with the structural stability, processability, and easy handling of alginate beads. Unlike raw sawdust, which is difficult to separate from treated water and prone to swelling and dispersion, immobilization within alginate enables the formation of mechanically stable, millimeter-scale beads that can be readily recovered. Compared to raw sawdust, the alginate introduces additional functional groups (e.g., hydroxyl (–OH) and carboxylate (–COO^−^)), thereby enhancing the interaction with cationic dye molecules. This synergistic combination allows the composite to act as a hybrid biosorbent in which both constituents contribute to dye uptake.

Therefore, this study aims to synthesize a composite biosorbent by immobilizing sawdust within a calcium alginate matrix and to investigate its biosorption performance toward cationic dyes from aqueous solutions, using malachite green as a representative model compound. Batch adsorption experiments were conducted to evaluate the effects of operational parameters on biosorption capacity. A mathematical modeling approach was employed to validate the experimental findings by applying various kinetic and equilibrium isotherm models, through which the kinetic parameters and equilibrium data associated with MG biosorption were determined and critically evaluated.

## 2. Materials and Methods

### 2.1. Materials, Reagents, and Analytical Procedure

**Sawdust.** The sawdust used in this study was collected from the industrial platform of the company WOODGRADE (Bacău, Romania), which specializes in wooden furniture manufacturing and exclusively processes oak and pine lumber as raw material. As a result, the sawdust is free from other wood residues such as bark or wood chips, ensuring a consistent and uncontaminated composition.

Multiple types of sawdust are generated on the platform, originating from various stages of the wood processing workflow. These types differ significantly in terms of particle size distribution, depending on the specific operation (e.g., cutting, milling, sanding).

**Figure 1:**
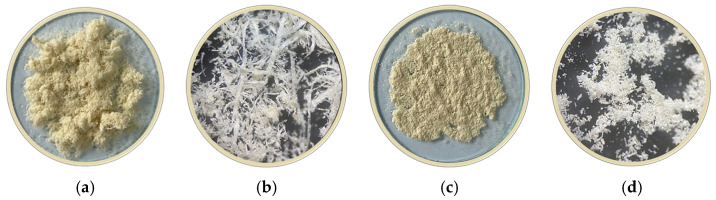
Images of the sawdust samples used in the experiments: (**a**) native image of the fine-grained sample collected from the platform (PSD); (**b**) microscope image of PSD; (**c**) native image of the sawdust sample used for biosorbent synthesis (PSDB); (**d**) microscope image of PSDB.

For the synthesis process, only fine sawdust (Figure 1a,b) produced during sanding and calibration operations was selected, due to its suitable granularity and homogeneity. This material is collected through filter bags integrated into the dust extraction system, which ensures high purity.

The fine sawdust sample was sieved, and the granulometric fraction (Figure 1c,d) consisting of particles with sizes between 0.050 mm and 0.100 mm was used in the synthesis process. Microscope images were obtained using an Optika ST-30FX stereo microscope (OPTIKA, Via Rigla, Italy).

**Sodium alginate**, a natural linear polysaccharide composed of α-L-mannuronic acid and *β*-D-guluronic acid units linked through 1,4-glycosidic bonds, is the sodium salt of alginic acid. It is primarily extracted from the cell walls of brown algae and exhibits chelating activity. Due to its biodegradability and biocompatibility, sodium alginate is widely used in the food, pharmaceutical, and cosmetic industries. In this study, sodium alginate was supplied by Sigma-Aldrich (Darmstadt, Germany), and its specifications, as provided by the manufacturer, are detailed in Table 1.

**Malachite green** dye used in this study was purchased from Merck (Darmstadt, Germany) and was of analytical grade. The characteristics of this dye, as provided by the manufacturer, are summarized in Table 2.

All solutions utilized in the experiments were prepared using distilled water, and pH was adjusted using NaOH (0.1 M) or HCl (0.1 M). A stock solution of malachite green (500 mg/L) was initially prepared and stored at 4 °C. Working solutions with concentrations ranging from 1 mg/L to 7 mg/L were subsequently prepared by dilution. Their absorbance was measured at 618 nm using a UV-1280 spectrophotometer (Shimadzu, Tokyo, Japan), and the resulting data were employed to construct the calibration curve. All the experiments were conducted in triplicate to ensure reproducibility.

### 2.2. Synthesis of Biocomposite Material

The synthesis of the composite material was carried out by immobilizing sawdust within a natural polymer matrix. For the preparation of the composite material, the granulometric fraction with particle sizes ranging between 0.050 and 0.100 mm was previously dried at 105 °C for 3 h and cooled to ambient temperature in a desiccator prior to use. A 1% (*w*/*v*) sodium alginate solution was prepared by dissolving sodium alginate in distilled water at 70 °C under magnetic stirring until complete dissolution. Dried sawdust was then incorporated into the alginate solution to a final concentration of 5% (*w*/*v*). The suspension was magnetically stirred for 4 h at ambient temperature to ensure homogeneity. The resulting suspension was dropwise into a 2% (*w*/*v*) calcium chloride solution to induce Ca^2+^ mediated ionic crosslinking of the alginate chains and form composite beads. The obtained beads were stored at 4 °C in calcium chloride solution until use.

### 2.3. Biocomposite Material Characterization

Fourier-transform infrared (FTIR) spectroscopy was employed to identify the functional groups involved in the biosorption mechanism. FTIR spectra of the biosorbent samples before and after biosorption were recorded using a Shimadzu IR Spirit-X spectrometer (Tokyo, Japan). The spectra were collected in the range of 400–4000 cm^−1^ using the attenuated total reflectance (ATR) mode.

The surface morphology and elemental composition of the biosorbent were examined using scanning electron microscopy (SEM) coupled with energy-dispersive X-ray spectroscopy (EDS) on a TESCAN MIRA S6122 microscope (TESCAN ORSAY HOLDING, Brno, Czech Republic). Precise navigation to the area of interest was ensured by the Wide Field Optics™ system, which provides a live SEM overview of the sample. This system replaces the traditional charge-coupled device (CCD) camera and offers unprecedented depth of focus and real-time visualization of the sample topography, enabling intuitive and accurate navigation. Prior to SEM–EDS analysis, the biosorbent beads were dried at 60 °C for 2 h. SEM images were acquired in secondary electron (SE) mode using an Everhart–Thornley detector under high-vacuum conditions. The operating parameters included an accelerating voltage of 5 kV (10 pA) and a working distance between 40.30 and 40.75 mm. The magnification range used for imaging was 10–500 µm. EDS analysis was performed at an accelerating voltage of 25 kV (30 pA), with a working distance between 20.30 and 20.48 mm and a magnification of 20 µm.

### 2.4. Determination of the Point of Zero Charge (pH_pzc_)

The point of zero charge (pH_pzc_) of the biosorbent was determined using the batch technique. To maintain a constant ionic strength throughout the experiment, a 0.1 M sodium chloride (NaCl) solution was utilized as the background electrolyte. A series of 20 mL NaCl solutions with initial pH values ranging from 2.0 to 12.0 was prepared by adjusting the pH using standardized 0.1 M hydrochloric acid (HCl) or 0.1 M sodium hydroxide (NaOH) solutions. Subsequently, 0.400 g of the biosorbent was introduced into each solution. The suspensions were subjected to continuous agitation at 130 rpm using an orbital shaker (model SK-O180-S, DLAB Scientific, Beijing, China) under ambient conditions (22 ± 1 °C) for a duration of 24 h. Following decantation of the supernatant, the final pH of each solution was measured. The variation in pH (ΔpH = pH_final_ − pH_initial_) was plotted as a function of the initial pH. The pH value at which ΔpH equals zero was identified as the point of zero charge (pH_pzc_) of the biosorbent. A similar approach was employed by Saadi et al. (2025) [46] for biosorbents derived from plant residues and by Mokhtari et al. (2021) [47] for 3D porous bioadsorbents based on chitosan/alginate/cellulose nanofibers.

### 2.5. Batch Biosorption Studies

The biosorption process of the MG dye was investigated using the batch mode. The effects of key parameters: pH, biosorbent dosage, and initial MG concentration on the biosorption process were evaluated. The experimental setup initially focused on examining the influence of the initial pH of MG solutions (20 mg/L), which was varied from 2 to 12, while the biosorbent dose was maintained at 3 g/L. The biosorbent dosages investigated ranged from 1 to 6 g/L. Additionally, the effect of increasing the initial MG concentration from 10 to 50 mg/L was studied.

For the kinetic study, 1.8 g of biosorbent was added to 30 mL of dye solution with varying initial dye concentrations (10, 20, 30, 40, and 50 mg/L) under constant agitation at 130 rpm and ambient temperature (21 ± 2 °C) for 360 min. At predetermined time intervals, the residual dye concentration in the supernatant was determined using a UV-Vis spectrophotometer (Shimadzu UV-1280, Tokyo, Japan). The MG concentrations were calculated based on a standard calibration curve constructed at λ_max_ = 618 nm. For the determination of biosorption capacity and removal efficiency, well-established equations widely recognized and frequently reported in the relevant scientific literature were employed [7,24,48]. Dye biosorption capacity (*q*) was determined through Equation (1):

(1) $$q_{t}\left(mg/g\right)=\frac{\left(C_{0}-C_{t}\right)}{m}\cdot V$$

where *q_t_* (mg/g), *C*_0_ (mg/L), and *C_t_* (mg/L) indicate the amount of adsorbed MG dye at time t, initial dye concentration, and dye concentration at time t, respectively. *V* (L) and *m* (g) represent the solution volume and the biosorbent dosage, respectively. The adsorption capacity at equilibrium (*q_e_*, mg/g) was calculated based on Equation (1) (by introducing the *C_e_* concentration at equilibrium instead of the *C_t_* concentration at time t). Equation (2) (shown below) was used to calculate the removal efficiency (*R*, %).

(2) $$R\left(\%\right)=\frac{C_{0}-C_{e}}{C_{0}}\cdot 100$$

### 2.6. Biosorption Kinetics and Isotherms

Adsorption kinetics controls the rate of uptake and the time required to reach equilibrium, playing a key role in process development and adsorption system design, and is commonly described using chemical reaction, diffusion-controlled, or mass-transfer-based kinetic models to elucidate adsorption mechanisms. Model fitting may be performed using linear or nonlinear approaches, with performance typically assessed using goodness-of-fit criteria, such as the coefficient of determination (R^2^), to identify the model that best represents the experimental data [49,50,51].

In an attempt to identify the rate-limiting steps and the processes governing MG biosorption onto the synthesized biosorbent, the experimental data obtained for the biosorption capacity *q_t_* (mg/g) as a function of contact time t (min) were analyzed using the nonlinear forms of the pseudo-first-order, pseudo-second-order, pseudo-n-order, Elovich, and Weber-Morris models, in order to determine which model best describes MG biosorption onto the SDA-5% biosorbent. Table 3 presents the individual equations, the significance of their parameters, and a short description of each model.

Adsorption isotherms define the equilibrium distribution of an adsorbate between the liquid phase and the adsorbent surface and are essential for evaluating adsorption capacity and mechanism. Plotting the equilibrium uptake (*q_e_*) as a function of the equilibrium concentration (*C_e_*) at constant temperature provides insight into adsorbent–adsorbate affinity, interaction strength, and the heterogeneity of adsorption sites. The isotherm profile reflects both the stability of these interactions and the ability of the adsorbent to remove specific species from solution. A wide range of models, including those of Langmuir, Freundlich, Redlich-Peterson, Temkin, Hill, and Toth, are routinely used to characterize adsorption behavior, from idealized monolayer adsorption to empirical multistep mechanisms [49,54].

In this work, the nonlinear Langmuir, Freundlich, Temkin, Hill, Redlich-Peterson, and Sips models were applied to describe MG biosorption onto the SDA 5% biosorbent.

Table 4 summarizes the equations of the selected isotherm models, the significance and units of their parameters, and a short model description.

## 3. Results and Discussion

### 3.1. Characterization of Synthesized Biosorbent

**Figure 2:**
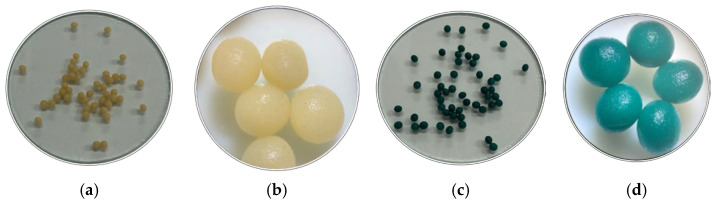
Images of the synthesized SDA 5% biocomposite granules before and after MG biosorption: (**a**) native image before biosorption; (**b**) stereomicroscopic image before biosorption; (**c**) native image after biosorption; (**d**) stereomicroscopic image after biosorption. Stereomicroscopic images were acquired using an Optika ST-30FX stereomicroscope (OPTIKA, Via Rigla, Italy) with 2× magnification and a WF 10×/20 mm ocular lens.

A new type of biosorbent, designated SDA 5%, was synthesized by immobilizing sawdust biomass in sodium alginate. This is a natural, non-toxic, biodegradable polymer derived from renewable resources and capable of forming stable crosslinked network structures in the presence of divalent cations such as calcium, making it an effective support material for biosorbent preparation. The appearance of the SDA 5% beads before and after MG biosorption is illustrated in Figure 2.

As shown, the synthesized biosorbent beads prior to biosorption display a light beige color and a uniform spherical shape, with an average diameter of 3.0760 ± 0.0700 mm.

#### 3.1.1. Fourier Transform Infrared Spectra (FTIR) Analysis

Fourier Transform Infrared (FTIR) spectroscopy was performed to identify the functional groups present on the surface of the biosorbent before and after biosorption, as these groups may play a significant role in the efficiency of MG dye removal.

**Figure 3:**
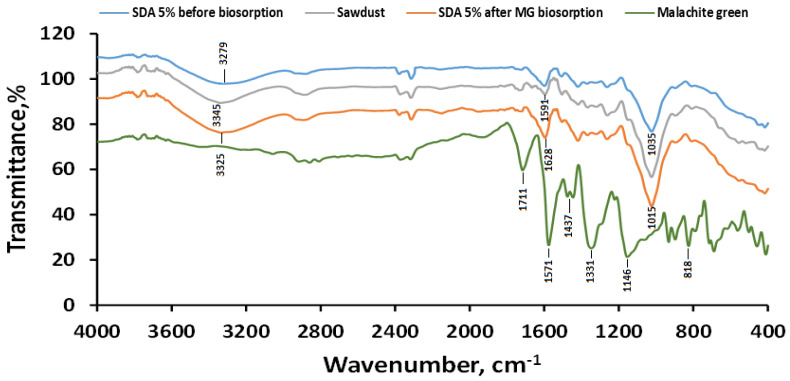
FTIR spectra of MG, sawdust, and SDA 5% biosorbent before and after malachite green biosorption.

The FTIR spectra of MG as the investigated dye molecule, the sawdust used in the synthesis as raw material, and the biosorbent before and after biosorption are presented in Figure 3.

The FTIR spectrum of the synthesized biosorbent SDA 5% primarily reveals the presence of functional groups characteristic of calcium alginate, along with those associated with the major components of sawdust: cellulose, hemicellulose, and lignin.

Broad absorption bands at high frequencies are observed at 3279 cm^−1^ for SDA 5% before biosorption, 3345 cm^−1^ for raw sawdust, and 3325 cm^−1^ for SDA 5% after MG biosorption. These bands correspond to O–H stretching vibrations involved in hydrogen bonding within cellulose, alginate, and related structures, in agreement with results reported by Makarov et al. (2025) [59] and Rao et al. (2023) [60]. It is also observed that the stretching vibrations of aliphatic C–H groups appear in the region of 2869–2925 cm^−1^.

A strong band at 1711 cm^−1^ indicates the stretching vibration of the C=N bond in MG. Additionally, an intense peak at 1571 cm^−1^ is associated with the aromatic C=C stretching in the phenyl rings of MG. Although a band in the 1600–1650 cm^−1^ region, corresponding to the asymmetric stretching vibration of the carboxylate (COO^−^) groups in alginate, would be expected, it cannot be clearly distinguished in the FTIR spectrum after biosorption due to overlap with other vibrational bands.

The bending vibration of the methyl C–H group in MG is detected at 1437 cm^−1^. MG-specific signals corresponding to C–N and C_ar_–N bond in tertiary aromatic and aliphatic amines were also identified, appearing at 1331 cm^−1^ (aromatic amines) and 1146 cm^−1^ (aliphatic amines), respectively. The presence of MG aromatic rings is further confirmed by the out-of-plane C–H bending vibration at 818 cm^−1^ within the fingerprint region, characteristic of *p*-substituted benzene rings. Similar observations were reported by Meskel et al. (2024) [61].

Weak peaks around 1254 cm^−1^ correspond to C–O stretching vibrations, which are typical of sawdust components [32]. The absorption band at 1406–1408 cm^−1^ is attributed to hemicellulose, while the intense peaks observed at 1015, 1029, and 1035 cm^−1^ are assigned to the symmetric and asymmetric stretching vibrations of C–O bonds in the pyranose ring of natural polymers [62].

A comparison of the spectra of SDA 5% before and after MG biosorption shows slight shifts in the characteristic peak positions relative to the initial biosorbent. These modifications indicate interactions between MG molecules and the functional groups of the biosorbent, confirming that the biosorption process has taken place.

#### 3.1.2. SEM and EDS Analysis

**Figure 4:**
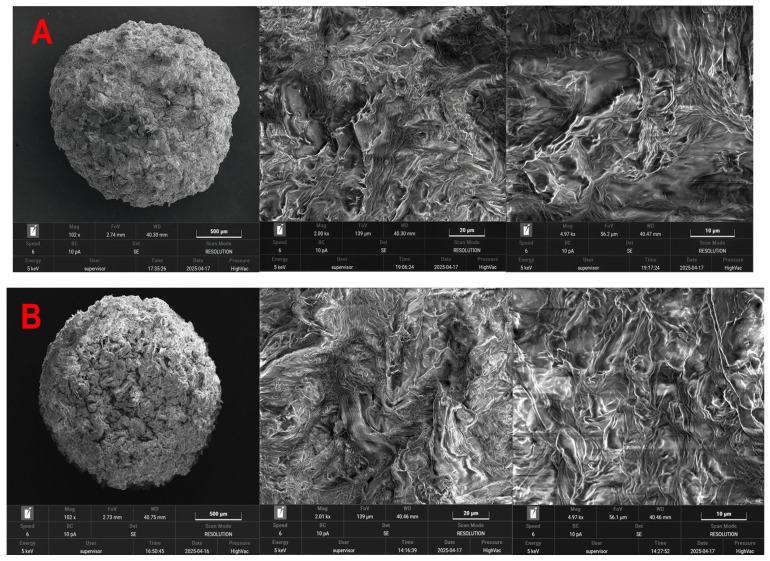
SEM images of SDA 5% biosorbent before (**A**) and after (**B**) malachite green biosorption.

SEM analysis (Figure 4) reveals significant morphological differences in the SDA 5% biocomposite before and after MG biosorption.

Prior to biosorption (Figure 4A), the material exhibits a highly porous surface with pronounced irregularities and open cavities, suggesting a high number of active sites available for adsorption. The micrographs also show a well-integrated distribution of sawdust within the alginate matrix, evidenced by the continuity of the phases and the absence of clear boundaries between components. This homogeneous structure confirms the effective embedding of sawdust into the polymer network, contributing to both composite stability and a uniform distribution of adsorption sites.

After MG biosorption (Figure 4B), the surface becomes visibly more compact, with pores and microcavities partially or completely filled by non-uniform deposits attributed to adsorbed dye molecules. The presence of aggregated domains and continuous surface layers further supports the formation of an adsorbed MG film across the material. These morphological changes result in a denser surface architecture and confirm the physicochemical interactions between the dye and the functional groups of the biosorbent. Similar surface changes in sawdust-based adsorbents during methylene blue dye adsorption were also reported by Ahamad et al. (2024) [11].

Overall, the evolution from the initially porous, well-distributed sawdust-alginate structure to the pore-blocked, aggregate-rich morphology after biosorption confirms both the successful synthesis of the SDA 5% composite and its high potential for MG removal via a mixed mechanism involving micropore retention and surface interactions.

**Figure 5:**
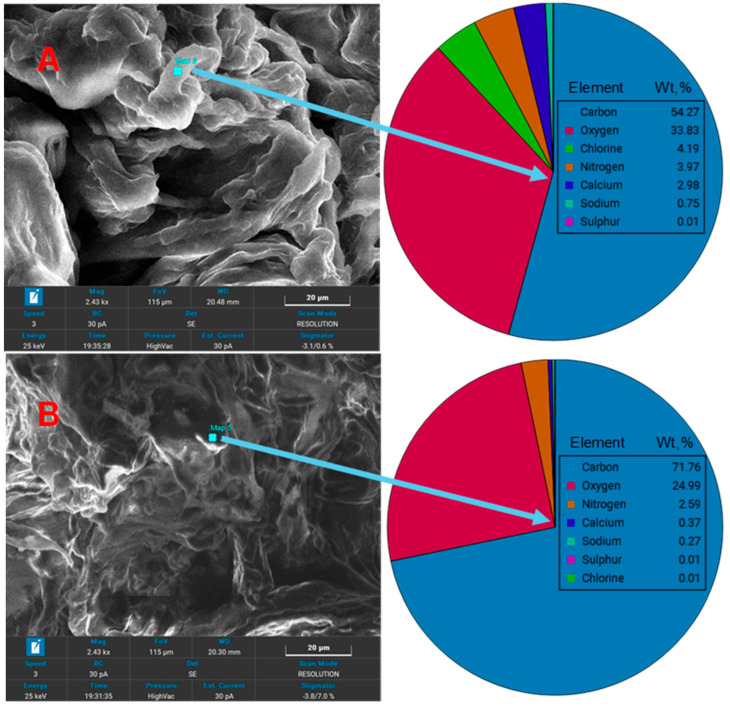
EDS analysis and elemental composition of SDA 5% biosorbent before (**A**) and after (**B**) malachite green biosorption.

The EDS analysis (Figure 5) shows that the SDA 5% biomaterial contains the elements C, O, N, Ca, Na, S, and Cl, a profile characteristic of a lignocellulosic biocomposite crosslinked with alginate.

In the sample before biosorption, C (54.27%) and O (33.83%) dominate the composition, reflecting the organic structure of sawdust and the polysaccharide groups present in alginate. Moreover, the relatively high percentage of Ca (2.98%) associated with the low Na content (0.75%) confirms the crosslinking process between alginate and sawdust, during which Ca^2+^ ions replace Na^+^ ions, thereby stabilizing the polymeric network.

After biosorption (Figure 5B), the elemental composition changes significantly: the increase in carbon (71.76%) and decrease in oxygen (24.99%) suggest the formation of an organic layer of dye deposited on the material surface. The reductions in N, Ca, and Na indicate the coverage of previously exposed sites or surface reorganization induced by the biosorption process. The very low Cl level in the final sample (0.01%) suggests either its masking by the deposited organic layer or a non-uniform distribution of the dye in the analyzed region. The increase in carbon content detected by EDS after malachite green adsorption is consistent with dye deposition on the biosorbent surface; however, EDS should be regarded as a localized, semi-quantitative technique. Surface charging effects, possible conductive coating, and the heterogeneous, porous nature of the biocomposite may influence elemental quantification, especially for light elements. Moreover, the dominance of the carbon-rich alginate and lignocellulosic matrix, along with the non-uniform distribution of adsorbed MG, can mask the relative contribution of nitrogen, leading to variations in the measured elemental percentages.

Changes in elemental composition associated with both composite synthesis and dye biosorption have also been reported in previous studies, including those of Ahamad et al. (2024) [11] for chemically modified *Azadirachta indica* sawdust used in methylene blue adsorption, and Vishwakarma et al. (2024) [63] for malachite green adsorption onto sodium alginate/bentonite nanocomposites.

In summary, the changes observed between the two EDS profiles confirm the surface modification of the biocomposite following MG biosorption, a process evidenced by the increase in the organic fraction and the reorganization of mineral elements, supporting a mixed adsorption mechanism involving physicochemical interactions and micropore retention.

#### 3.1.3. Point of Zero Charge (pH_pzc_)

The point of zero charge (pH_pzc_) represents the pH at which the surface of an adsorbent carries no net charge, and it was determined for the synthesized SDA 5% biosorbent using the pH_pzc_ drift method. The pH_pzc_ was evaluated over a pH range of 2–12. During the experiments, major morphological changes of the granules were observed at extreme pH values: at pH 2, the granules shrank; at pH 12, they increased in diameter and showed signs of structural degradation.

**Figure 6:**
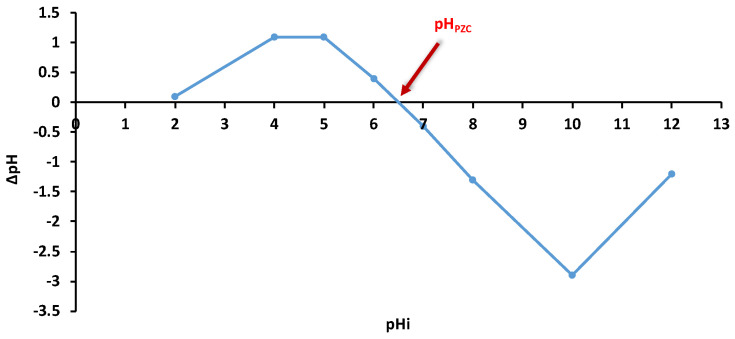
Determination of pH_pzc_ using the pH drift method for SDA 5%.

As shown in the pH_pzc_ plot (Figure 6), the SDA 5% biosorbent exhibits a point of zero charge at 6.5, indicating that the surface becomes neutrally charged at this value.

A comparable pH_pzc_ value (6.8) was reported by Ahamad et al. (2024) [11] for NaOH-modified *Azadirachta indica* sawdust.

### 3.2. Study of Operational Parameters

#### 3.2.1. Effect of pH on MG Dye Removal

The pH is a critical parameter governing biosorption efficiency, as it influences the degree of ionization of functional groups, the surface charge of micropores, and the chemical speciation of the dye. The effect of pH on MG removal by SDA 5% was investigated over a broad pH range (2–12) at an initial dye concentration of 20 mg/L. During preliminary tests, a distinct pH-dependent color change of the MG solution was observed: partial discoloration occurred at pH 2, the solution remained intensely colored between pH 3–6, gradually shifted to lighter shades in the pH 7–10 range, and became almost colorless at pH 12, indicating significant instability of MG under strongly alkaline conditions. UV–Vis spectra of Malachite green recorded over a wide pH range (2–12) are provided in the Supplementary Materials, highlighting pH-induced spectral variations and dye stability. Since the structural stability and chromatic integrity of malachite green are preserved only under acidic conditions, all subsequent biosorption experiments were carried out within the pH range of 3–6, where the dye remains stable and retains its characteristic blue coloration.

Azaman et al. (2018) [7] similarly reported that the MG solution becomes colorless under alkaline conditions.

**Figure 7:**
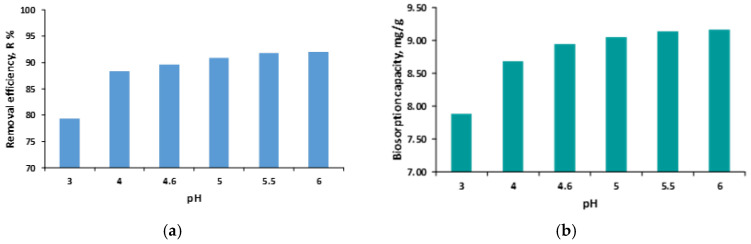
Influence of the initial pH of the MG solution on biosorption performance, expressed as removal efficiency (**a**) and biosorption capacity (**b**). (experimental conditions: pH range: 3–6; initial MG concentration: 20 mg/L; biosorbent dose: 2.5 g/L; agitation speed: 130 rpm).

The results illustrating the influence of pH on biosorption capacity and removal efficiency are shown in Figure 7.

As shown in Figure 7, the highest MG removal was achieved between pH 5 and 6 (including pH 5.5), where the biosorbent exhibited removal efficiencies of 90.9–92.0% and biosorption capacities of 9.05–9.16 mg/g. In contrast, significant decreases in both removal efficiency and adsorption capacity were recorded at pH 3, indicating that highly acidic conditions hinder MG uptake. Based on these findings, pH 6 was selected for the subsequent experiments not only because it yielded superior removal efficiency and biosorption capacity, but also because working at this pH is advantageous for practical applications. Most natural water systems fall within a pH range of 5.5–8.5, meaning that operating at pH 6 minimizes the need for additional pH adjustment. This choice ensures that the adsorption experiments are performed under conditions that closely reflect real-world water treatment environments.

Supporting evidence is provided by Singh et al. (2018) [64], who conducted MG adsorption at pH 6 using *Mangifera indica* seed powder and reported a removal efficiency of 96% at an initial concentration of 100 mg/L.

#### 3.2.2. Effect of Biosorbent Dose

**Figure 8:**
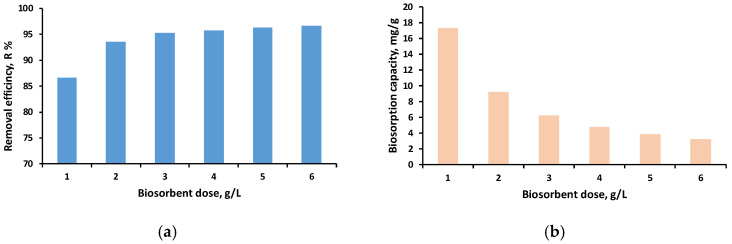
Influence of the biosorbent dose on the MG solution on biosorption performance, expressed as removal efficiency (**a**) and biosorption capacity (**b**). (experimental conditions: pH 6; initial MG concentration: 20 mg/L; biosorbent dose range: 1–6 g/L; agitation speed: 130 rpm).

The adsorbent dosage plays a key role in biosorption performance, as it determines the efficiency with which contaminants are removed. In this study, SDA 5% was tested at dosages ranging from 1.0 to 6.0 g/L, and the corresponding results are presented in Figure 8.

As shown in Figure 8a, SDA 5% achieves high removal efficiencies between 95.31% and 96.61% for adsorbent doses of 3, 4, 5, and 6 g/L, indicating that within this range the removal percentage does not vary significantly. In contrast, lower doses of 1 and 2 g/L result in noticeably reduced removal performance.

Figure 8b shows that the biosorption capacity decreases as the adsorbent dose increases, a trend that can be explained by the rapid saturation of available active sites and by the fact that, at higher dosages, the amount of dye in solution becomes insufficient relative to the large surface area provided by the added material, resulting in less dye adsorbed per unit mass.

Based on these observations, a dose of 3 g/L was selected for subsequent experiments, as higher doses do not provide meaningful improvements in either removal efficiency or biosorption capacity.

#### 3.2.3. Effect of MG Initial Concentration

Evaluating a new biosorbent involves determining how its adsorption capacity varies with the initial dye concentration, as both concentration gradients and available active sites on the biosorbent surface strongly influence biosorption performance [25,65].

**Figure 9:**
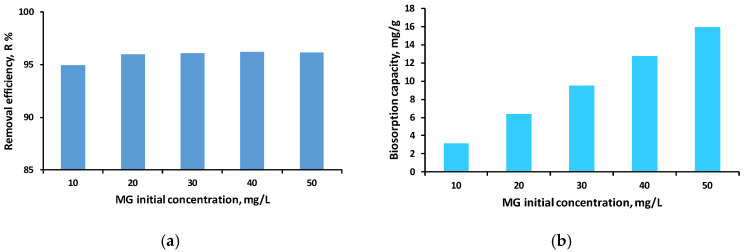
Influence of MG various initial concentration solutions on biosorption performance, expressed as removal efficiency (**a**) and biosorption capacity (**b**); (experimental conditions: pH 6; initial MG concentration range: 10–50 mg/L; biosorbent dose: 3 g/L; agitation speed: 130 rpm).

The influence of the initial MG concentration was assessed under constant pH, shaking speed, and biosorbent dosage, with the corresponding results presented in Figure 9.

Figure 9a shows that the removal efficiency remains high and relatively constant across the entire range of tested concentrations (10–50 mg/L), with values between 94.92% and 96.18%. This stability suggests that SDA 5% provides a sufficient number of active sites to effectively retain MG even at higher concentrations, without a significant decrease in removal performance.

In contrast, Figure 9b indicates a clear and progressive increase in biosorption capacity as the initial dye concentration increases. This trend is typical for adsorption systems involving solid materials, as higher concentrations enhance the diffusion gradient and the availability of MG molecules, resulting in greater loading of the biosorbent surface. Thus, each gram of biosorbent retains more dye at higher initial concentrations, even though the overall removal percentage remains nearly constant.

Comparable findings were reported by Azaman et al. (2018) [7], who noted that the adsorption rate increases with rising initial dye concentration during the removal of malachite green using coconut-shell activated carbon as adsorbent.

Taken together, the two graphs suggest that SDA 5% performs efficiently across a wide range of initial dye concentrations, maintaining a high removal efficiency while the biosorption capacity increases proportionally with the initial dye availability. This behavior indicates a biosorption mechanism strongly dependent on the concentration gradient.

### 3.3. Biosorption Kinetics

The kinetic analysis involved fitting the experimental data to nonlinear forms of five widely used models: pseudo-first-order, pseudo-second-order, pseudo-n-order, Elovich, and Weber–Morris, to identify the model that best describes MG biosorption onto the SDA 5% biosorbent under the selected operating conditions. Table 5 summarizes the kinetic model parameters obtained for the tested MG concentrations across all evaluated models.

**Figure 10:**
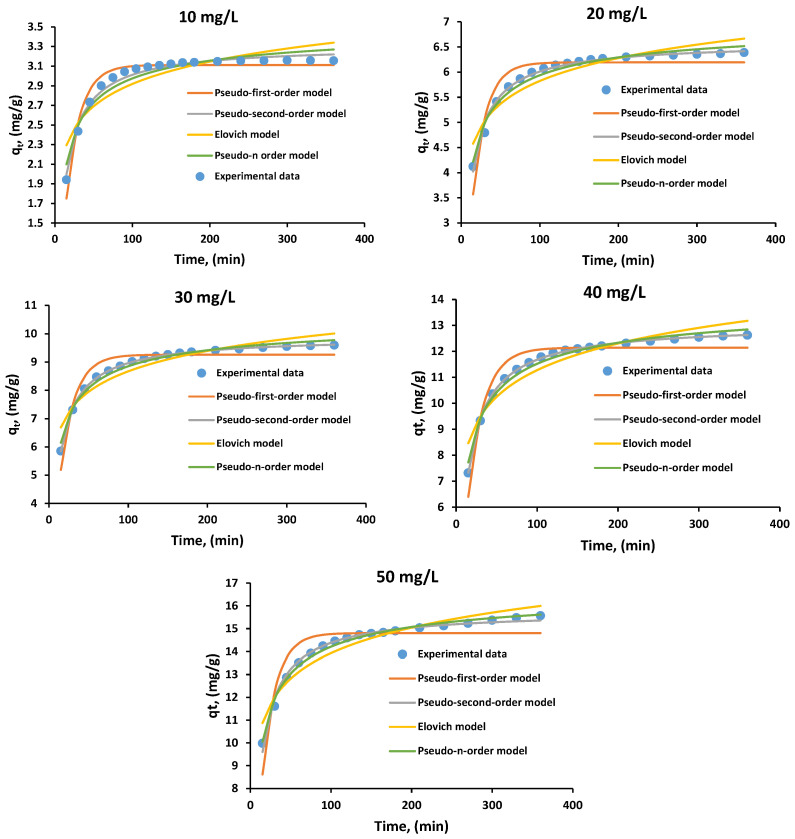
Nonlinear kinetic model fitting for MG biosorption onto SDA 5% at initial MG concentrations of 10–50 mg/L, compared with experimental data. Experimental conditions: pH 6, biosorbent dose 3 g/L, shaking speed 130 rpm, contact time 360 min.

The pseudo-second-order and pseudo-n-order models show the closest agreement with the experimental data. Among them, the pseudo-second-order model provides the best overall fit, with correlation coefficients ranging from 0.9807 to 0.9996 for all tested concentrations. Figure 10 illustrates the fitted kinetic models in comparison with the experimental data obtained for the biosorption process.

The kinetic results indicate that MG biosorption onto SDA 5% is well described by the pseudo-second-order model, which accurately captures the experimental adsorption behavior over the investigated time range. The excellent agreement with this kinetic model suggests an appropriate description of the rate-controlling steps in the biosorption process. However, it should be noted that such a fit does not constitute direct evidence of chemisorption. Instead, the observed kinetic behavior is consistent with a complex adsorption mechanism involving multiple concurrent interactions, including hydrogen bonding, π–π and n–π interactions, electrostatic contributions, and pore-diffusion effects, as discussed in the proposed adsorption mechanism. The rapid initial uptake followed by the establishment of equilibrium reflects the availability of accessible active sites and the progressive occupation of both surface and internal pores of the biocomposite, rather than the exclusive formation of chemical bonds. The lower *k*_2_ values obtained at higher MG concentrations (40 and 50 mg/L) suggest that these systems require longer contact times to reach equilibrium. Moreover, the equilibrium adsorption capacities (*q_e_*) predicted by the pseudo-second-order model closely matched the experimental data for all concentrations, confirming its suitability for describing MG biosorption onto SDA 5%. Nonetheless, the selection of a kinetic model ultimately depends on the specific characteristics of the adsorbent–adsorbate system and the experimental conditions. A good fit to the pseudo-second-order model does not inherently imply superiority over other systems. It simply reflects the best mechanistic representation for this particular adsorption process.

Several other studies have likewise identified the pseudo-second-order model as the most suitable kinetic model for describing MG adsorption, including investigations using sodium carbonate–treated rice husk [3], coconut-shell activated carbon [7], *Rumex abyssinicus*-derived activated carbon [27], and rattan sawdust [1] as adsorbents.

### 3.4. Biosorption Isotherms

Isotherm models play an essential role in characterizing the interaction between biosorbents and adsorbates, offering insight into the adsorption mechanism and the overall sorption capacity of the material. Determining the most suitable adsorption equilibrium correlations, as well as the appropriate isotherm and kinetic models, is crucial for understanding the behavior of newly developed biosorbents and for achieving optimal adsorption performance [66]. The study of these mathematical models enables reliable prediction of adsorption parameters and constants and provides a solid basis for quantitative comparisons between different adsorbents, adsorption systems, or experimental conditions.

Adsorption isotherms describe how pollutants interact with adsorbent surfaces, serving as key tools for optimizing adsorption pathways and evaluating surface properties such as homogeneity or heterogeneity, as well as distinguishing between physical and chemical adsorption. By correlating experimental data with these models, the adsorption capacity can be determined accurately and compared with theoretical predictions [66,67].

Ultimately, applying these models allows the establishment of an equilibrium relationship between the biosorbent (SDA 5%) and the adsorbate (MG dye), contributing to a deeper understanding of the adsorption process.

Considering that several studies have reported significant limitations of the linearized isotherm models, including their tendency to negatively affect data accuracy and introduce potential errors that may distort the design of an efficient adsorption system [66,68,69], the nonlinear approach was selected in this work. To avoid these drawbacks and ensure reliable data comparability, the experimental results for MG biosorption onto SDA 5% were fitted using the nonlinear forms of the Langmuir, Freundlich, Redlich–Peterson, Temkin, Hill, and Sips models, according to the equations presented in Table 4 (Materials and Methods Section).

**Figure 11:**
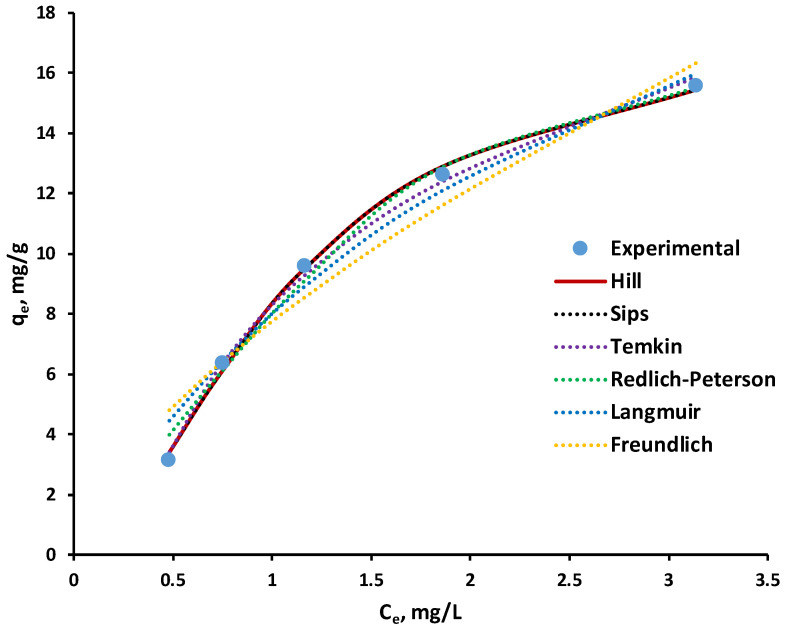
Nonlinear fitting of equilibrium isotherm models to the experimental MG biosorption data onto SDA 5% biosorbent. Experimental conditions: initial MG concentration 10–50 mg/L, pH 6, biosorbent dose 3 g/L, shaking speed 130 rpm, contact time 360 min.

The results obtained for the tested biosorption isotherm models are illustrated in Figure 11, while the corresponding calculated isotherm parameters are summarized in Table 6.

The selection of the most appropriate isotherm model was based primarily on the correlation coefficient (R^2^), whose value is expected to approach unity for an optimal fit. The R^2^ parameter reflects the degree to which the experimental data conform to the theoretical model; values close to 1 indicate a high level of agreement and demonstrate that the model provides an accurate representation of the adsorption behavior.

The analysis of the parameters presented in Table 6, together with the graphical comparison shown in Figure 11, demonstrates that the tested isotherm models differ substantially in their ability to describe the biosorption of MG onto the SDA 5% biosorbent. The correlation coefficients (R^2^) indicate that the Hill (R^2^ = 0.9978) and Sips (R^2^ = 0.9978) models provide the most accurate representation of the experimental data, a conclusion visually supported by the almost perfect overlap between their predicted curves and the experimental points across the entire equilibrium concentration range. The excellent agreement of the experimental data with the Hill and Sips isotherm models suggests the presence of cooperative adsorption effects during MG biosorption onto SDA 5%. This behavior is attributed to the structurally and chemically heterogeneous nature of the biocomposite surface, which comprises functional groups derived from both alginate and lignocellulosic components. As adsorption proceeds, interactions between previously adsorbed MG molecules and incoming dye species, together with local changes in surface energy, can enhance the affinity of neighboring active sites. The resulting coverage-dependent interactions give rise to the sigmoidal adsorption behavior captured by both models and are consistent with the proposed biosorption mechanism involving hydrogen bonding, π–π and n–π interactions, and pore-filling processes within a heterogeneous adsorbent matrix.

The Temkin model also exhibits excellent agreement with the experimental data (R^2^ = 0.9968), particularly in the medium concentration region, confirming that adsorption energy decreases linearly with surface coverage as the model predicts.

The Redlich–Peterson model, which incorporates features of both the Langmuir and the Freundlich equations, also performs very well, yielding a high R^2^ value (0.9890). Its curve closely follows the experimental data over most of the concentration range, indicating that the adsorption process involves both homogeneous and heterogeneous adsorption sites, an aspect effectively captured by this hybrid model. Although the Redlich–Peterson model displays slightly lower accuracy compared with Hill, Sips, and Temkin, it remains a great descriptor of the adsorption behavior, reflecting the complexity of the MG–biosorbent interaction.

In contrast, the classical Langmuir model (R^2^ = 0.9730) deviates noticeably from the experimental data at higher concentrations, slightly overestimating the maximum adsorption capacity *q_e_*. This suggests that the biosorption process is not purely monolayer and that the biosorbent surface exhibits heterogeneity. The Freundlich model shows the largest deviations (R^2^ = 0.9437), especially at higher concentrations, underestimating *q_e_* and confirming its limitations under high-coverage conditions.

Overall, both the numerical parameters and graphical trends indicate that the Hill and Sips models, followed closely by the Temkin model, provide the most reliable description of MG biosorption onto SDA 5%. The constants derived from these models highlight a high affinity toward MG, along with progressive, structure-dependent, and cooperative adsorption behavior. The Redlich–Peterson model, with its high R^2^ value, further supports the presence of a complex adsorption mechanism.

These findings suggest that MG biosorption onto SDA 5% follows a mixed mechanism, governed by adsorbent-adsorbate interactions involving contributions from both uniform monolayer regions and heterogeneous adsorption sites, thereby explaining the high performance and stability of this biosorbent.

Several studies investigating dye adsorption onto various adsorbent materials have reported that experimental adsorption data often exhibit a better fit with different isotherm models, depending on the nature and physicochemical properties of the adsorbent [7,67,70,71,72,73]. For example, Filho et al. (2023) [67], in their study on the removal of Malachite Green and Congo Red dyes using wastes from mining and processing perlite, found that the adsorption of both dyes was best described by the Sips isotherm model. Similarly, Azaman et al. (2018) [7] reported that MG adsorption onto coconut shell activated carbon was well represented by the Langmuir, Freundlich, and Temkin models, highlighting the combined influence of monolayer adsorption and surface heterogeneity. In contrast, the biosorption of Malachite Green using resting and immobilized *Bacillus cereus* biomass was most accurately fitted by the Redlich–Peterson model, indicating a mixed adsorption mechanism. Another study on the biosorption of Crystal Violet using the aerial parts of *Chenopodium album* showed that the equilibrium data were best fitted by the Freundlich and Hill models, based on the highest regression coefficients obtained.

In summary, these findings emphasize that the most appropriate isotherm model depends strongly on the structural characteristics of the adsorbent and the specific interactions occurring between the dye molecules and the adsorbent surface.

### 3.5. Possible Biosorption Mechanism

**Figure 12:**
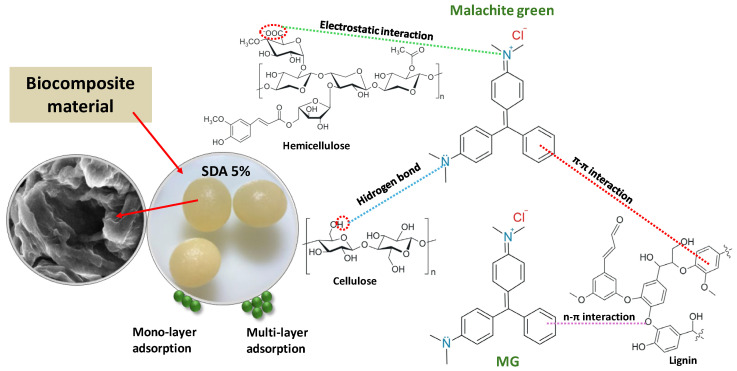
Proposed interaction of MG dye with SDA 5% biocomposite material.

The potential biosorption mechanism illustrated in Figure 12 provides insight into the fundamental interactions governing the removal of malachite green (MG) dye by the SDA 5% biocomposite material. The process primarily involves the attachment of MG dye molecules onto the surface and within the porous structure of the biocomposite, leading to the effective extraction of the dye from the aqueous solution. Experimental results showed that MG removal was more efficient under weakly acidic conditions (solution pH < pH_pzc_). According to pH drift measurements, the pH_pzc_ value of SDA 5% is 6.5, indicating that, within the investigated pH range (3–6), the biosorbent surface is predominantly positively charged. Since MG is a cationic dye, purely electrostatic attraction between the dye molecules and the biosorbent surface is not expected to be the dominant driving force. However, the enhanced removal efficiency observed at pH 5–6 suggests that the adsorption process is governed by a combination of interaction mechanisms rather than by electrostatic effects alone. Under these conditions, electrostatic interactions may still contribute indirectly through interactions with negatively charged counter ions or localized negatively charged functional sites. This interpretation is supported by the appearance of additional absorption bands in the FTIR spectra after biosorption (Figure 3), indicating the involvement of surface functional groups and changes in their chemical environment following MG uptake.

As schematically mechanism shown in Figure 12, the SDA 5% biocomposite contains a wide range of functional groups originating from both sawdust components (cellulose, hemicellulose, and lignin) and sodium alginate. These constituents provide abundant adsorption sites such as hydroxyl (–OH), carboxyl (–COOH/–COO^−^), and ether groups. The coexistence of these functional groups enables multiple non-electrostatic interaction mechanisms.

In particular, hydrogen bonding can occur between the hydroxyl groups of cellulose and hemicellulose and the heteroatoms present in the MG molecule. Additionally, the presence of lignin in the SDA 5% matrix promotes π–π interactions between the aromatic structures of lignin and the conjugated aromatic rings of MG. These interactions are relatively insensitive to surface charge and become particularly relevant under pH conditions where electrostatic repulsion may be present. Such π–π stacking interactions contribute significantly to dye retention on the biosorbent surface and help explain the high adsorption efficiency observed near neutral pH values.

Alongside π–π interactions, n–π interactions may also occur between electron donor lone pairs (n) from oxygen atoms in hydroxyl or ether groups of cellulose, hemicellulose, and alginate, and the π electron system of the aromatic rings of MG. These n–π interactions further stabilize the adsorption complex and enhance dye uptake, particularly at near-neutral pH values. Beyond surface interactions, the adsorption mechanism is further enhanced by the porous morphology of the biocomposite, as illustrated by the SEM features in Figure 12. The presence of internal pores and surface roughness facilitates physical adsorption through pore diffusion, allowing MG molecules to penetrate into the interior of the biosorbent matrix. This structural characteristic supports both monolayer adsorption on accessible external surfaces and multilayer adsorption within the pore network, increasing overall adsorption capacity.

Overall, the biosorption of MG onto SDA 5% is governed by a synergistic mechanism involving electrostatic effects, hydrogen bonding, π–π interactions, and n–π interactions, as well as pore filling processes. Among these, non-electrostatic interactions and physical adsorption within the porous structure play a dominant role under weakly acidic conditions. The combined contribution of these mechanisms, together with the heterogeneous composition and developed porosity of the biocomposite material, accounts for the high MG removal efficiency observed experimentally.

Previous studies on Malachite green removal using biomass-derived and cellulose-based adsorbents, including cellulose extracted from waste bamboo and other biowaste materials, indicate that adsorption is generally governed by a multifaceted and synergistic mechanism involving hydrogen bonding, π–π and n–π interactions, pore-filling phenomena, and secondary electrostatic effects, rather than by a single dominant interaction [74,75]. Such behavior is characteristic of heterogeneous biobased adsorbent surfaces and is in good agreement with the adsorption mechanism proposed in the present study.

## 4. Conclusions

In this study, a novel biosorbent prepared by immobilizing sawdust in a calcium alginate matrix (SDA 5%) demonstrated high efficiency for the removal of Malachite Green (MG) from aqueous solutions. The spherical biosorbent beads (3.0760 ± 0.0700 mm; pH_pzc_ = 6.5) exhibited good structural stability during storage and were thoroughly characterized through SEM, EDS, and FTIR analyses. FTIR spectra revealed slight shifts in characteristic functional groups after biosorption, confirming interactions between MG and the biosorbent surface. SEM images showed a transition from an initially porous sawdust–alginate composite to a pore-blocked, aggregate-rich surface after MG uptake, while EDS analysis indicated changes in elemental composition consistent with surface modification and dye retention.

Operational parameters studies showed that high removal efficiencies (≥94.92%) were achieved under optimized conditions (pH 6, 3 g/L biosorbent dose, 21 ± 2 °C, 130 rpm) for MG concentrations ranging from 10 to 50 mg/L.

Kinetic analysis using nonlinear models demonstrated that the pseudo-second-order equation best described the adsorption process (R^2^ = 0.9807–0.9996), indicating chemisorption as the rate-limiting step. Likewise, equilibrium modeling performed in nonlinear form revealed that the Hill and Sips isotherm models, followed closely by Temkin, provided the most accurate fit to experimental data, highlighting the high affinity toward MG and suggesting a progressive, cooperative adsorption mechanism.

The good performance of the Redlich-Peterson model further supported the presence of a complex adsorption mechanism involving both uniform monolayer regions and heterogeneous adsorption sites.

In summary, SDA 5% proved to be an efficient, low-cost, and environmentally friendly biosorbent, offering significant potential for dye removal from contaminated effluents. The use of sawdust, a readily available resource, underscores the material’s economic sustainability and its potential contribution to circular bioeconomy practices.

Future research should focus on evaluating regeneration capacity, reusability, and scale-up feasibility to support its practical implementation in real wastewater treatment systems.

## Figures and Tables

**Table 1:** Sodium alginate characteristics.

Characteristics	Sodium Alginate
Structural formula	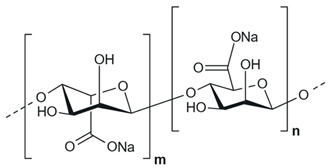
Linear formula	(C_6_H_7_NaO_6_)n
CAS No.	9005-38-3
Origin of material	Brown algae

**Table 2:** Chemical and structural properties of Malachite green dye.

Characteristics	Malachite Green
Structural formula	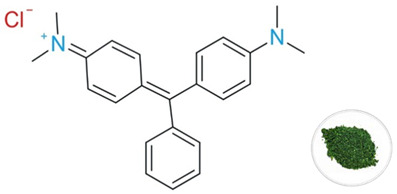
Linear formula	C_23_H_25_N_2_Cl
CAS No.	569-64-2
Molecular weight [g·mol^−1^]	364.911
λ_max_ [nm]	618
Nature	Basic (cationic) dye
Class	Triphenylmethane dye
Color	Dark green (solid)

**Table 3:** Nonlinear equations of kinetic models.

Kinetic Model	Equation	Parameter Significance	Ref. *
Pseudo- first-order	$q_{t}=q_{e}\cdot \left(1-e^{{-k}_{1}\cdot t}\right)$	*t*—time (min), *q_t_*—concentration on the solid phase at time *t* (mg/g), *q_e_*—adsorbent capacity at equilibrium (mg/g), *k*_1_—constant rate (1/min)	[49,50]
Model description	The pseudo-first-order (PFO) model is commonly applied to adsorption from aqueous solutions and assumes that the adsorption rate is proportional to the number of available binding sites. It is mainly valid for the initial stage of adsorption, particularly when interfacial diffusion controls the uptake process.		
Pseudo- second-order	$q_{t}=\frac{k_{2}\cdot q_{e}^{2}\cdot t}{\left(1+k_{2}\cdot q_{e}\cdot t\right)}$	*t*—time (min), *q_t_*—concentration on the solid phase at time *t* (mg/g), *q_e_*—adsorbent capacity at equilibrium (mg/g), *k*_2_—constant rate (g/(mg∙min))	[49,50,52]
Model description	The pseudo-second-order (PSO) kinetic model assumes that adsorption is governed by chemical interactions between the adsorbate and functional groups on the adsorbent surface and that the adsorption capacity is determined at equilibrium. It is commonly used to describe chemisorption processes and to estimate sorption capacity.		
Elovich	$q_{t}=\frac{1}{\beta }\cdot ln\left(1+\alpha \cdot \beta \cdot t\right)$	*t*—time (min), *q_t_*—concentration on the solid phase at time *t* (mg/g), *α*—initial adsorption rate (mg/(g∙min)), *β*—extent of surface coverage and activation energy for chemisorption (mg/g)	[49,50,52]
Model description	The Elovich model is an empirical kinetic model widely used to describe chemisorption on heterogeneous surfaces. It is based on the idea that adsorption involves increasing activation energy over time and occurs on energetically non-uniform sites. Although not derived from strict physical principles, it effectively represents adsorption systems with variable activation energies and often resembles second-order kinetics. First developed for gas–solid chemisorption, the Elovich equation is now widely used to describe the adsorption of pollutants from aqueous solutions due to its suitability for heterogeneous surfaces.		
Weber-Morris	$q_{t}=k_{wm}\cdot t^{0.5}+B$	*t*—time (min), *q_t_*—concentration on the solid phase at time *t* (mg/g), *k_wm_*—the intraparticle diffusion rate (mg/(g∙min^0.5^), *B*—constant rate (mg/g)	[52]
Model description	The Weber-Morris model is commonly applied to evaluate the contribution of intraparticle diffusion to the overall adsorption rate. In this approach, a linear relationship between qt and t^0.5^ indicates that intraparticle diffusion is the dominant mass-transfer step, whereas deviations from linearity or the appearance of multiple linear segments imply the involvement of several rate-limiting mechanisms. Adsorption on porous solids generally proceeds through four sequential steps: (i) transport of solute molecules from the bulk solution to the external boundary layer, (ii) diffusion across the boundary layer to the adsorbent surface, (iii) migration through macro- and micropores toward internal active sites, and (iv) interaction of the solute with these sites through processes such as adsorption, complexation, or precipitation.		
Pseudo-n- order	$q_{t}=q_{e}\left(1-\frac{1}{{\left[1+\left(n-1\right)\cdot q_{e}^{\left(n-1\right)}\cdot k_{n}\cdot t\right]}^{\frac{1}{\left(n-1\right)}}}\right)$	*t*—time (min), *q_t_*—concentration on the solid phase at time *t* (mg/g), *q_e_*—adsorbent capacity at equilibrium (mg/g), *k_n_*—constant rate (*q*^*n*−1^/(mg^1−n^∙min)) n_order_—(dimensionless)	[50,53]
Model description	The pseudo-n-order (PNO) model is an empirical kinetic equation used to describe adsorption processes in which the reaction order lies between 1 and 2 or even exceeds 2. By introducing a variable reaction order, it extends the applicability of the traditional PFO and PSO models and provides greater flexibility in representing complex kinetic mechanisms. Despite this advantage, the PNO model lacks explicit physical meaning, and its nonlinear form cannot be transformed into a linear expression, making its mathematical solution more complex than that of PFO and PSO. Even so, the model allows a more accurate interpretation of adsorption in heterogeneous systems and facilitates the precise determination of kinetic parameters under diverse conditions.		

Ref. *—references for each mentioned model.

**Table 4:** Nonlinear equation of investigated equilibrium isotherms.

Equilibrium Isotherm	Equation	Parameter Significance		Ref. *
Langmuir	$q_{e}=\frac{q_{l}\cdot K_{l}\cdot C_{e}}{1+K_{l}\cdot C_{e}}$	*q_e_*—adsorbate concentration on the solid phase at equilibrium (mg/g), *C_e_*—adsorbate concentration on the fluid phase at equilibrium (mg/L), *K_l_*—Langmuir constant (L/mg), *q_l_*—Langmuir constant (mg/g)		[54]
Model description	The Langmuir isotherm, originally developed for gas–solid adsorption on activated carbon, is widely used to evaluate and compare the performance of different biosorbents. It assumes monolayer adsorption on a finite number of identical, energetically equivalent sites, with no interaction effects between adsorbed molecules. The model also presumes constant adsorption energy across the surface and no surface mobility of the adsorbate molecules. The resulting isotherm exhibits a saturation plateau, indicating the point at which all sites are occupied and further adsorption is not possible.			
Freundlich	$q_{e}=K_{fr}\cdot C_{e}^{\frac{1}{n_{fr}}}$		*q_e_*—adsorbate concentration on the solid phase at equilibrium (mg/g), *C_e_*—adsorbate concentration on the fluid phase at equilibrium (mg/L), *K_fr_*—Freundlich constant ((mg/g)∙(L/mg)^1/*n*^_*fr*_), *n_fr_*—(dimensionless)	[49,54,55]
Model description	The Freundlich isotherm describes non-ideal, reversible, multilayer adsorption on heterogeneous surfaces, where adsorption sites exhibit a broad distribution of binding energies. As an empirical model, it assumes that high-energy sites are occupied first, followed by progressively weaker sites, producing an exponential decline in adsorption energy with increasing surface coverage. Initially developed for adsorption on animal charcoal, the model accounts for systems in which the adsorbed amount varies non-proportionally with solute concentration. Owing to its flexibility, the Freundlich isotherm is widely applied to heterogeneous adsorption processes, particularly for organic compounds and highly interactive species on activated carbon and molecular sieves.			
Temkin	$q_{e}=\frac{R\cdot T}{b_{t}}\cdot ln\left(K_{t}\cdot C_{e}\right)$	*q_e_*—adsorbate concentration on the solid phase at equilibrium (mg/g), *C_e_*—adsorbate concentration on the fluid phase at equilibrium (mg/L), *R*—gas constant (R = 8.314 J/(mol∙K)), *T*—temperature (K), *K_t_*—Temkin constant (L/mg), *b_t_*—Temkin constant (J/mg)		[54,55]
Model description	The Temkin isotherm was initially developed to describe hydrogen adsorption on platinum electrodes in acidic media. This model incorporates the effects of adsorbent-adsorbate interactions by assuming that the heat of adsorption decreases linearly with surface coverage once extremely low and high concentration ranges are excluded. Its formulation implies a relatively uniform distribution of binding energies up to a maximum value, distinguishing it from models based on logarithmic energy variation.			
Hill	$q_{e}=\frac{q_{hi}\cdot C_{e}^{n_{hi}}}{K_{hi}+C_{e}^{n_{hi}}}$	*q_e_*—adsorbate concentration on the solid phase at equilibrium (mg/g), *C_e_*—adsorbate concentration on the fluid phase at equilibrium (mg/L), *q_hi_*—Hill maximum uptake (mg/g), *K_hi_*—Hill constant (L/mg), n_hi_—(dimensionless)		[56,57]
Model description	The Hill isotherm equation, derived from the non-ideal competitive adsorption model, describes the binding behavior of multiple species on homogeneous substrates. This model conceptualizes adsorption as a cooperative phenomenon, whereby the interaction of a ligand or adsorbate at one binding site affects the affinity of additional sites on the same adsorbent or macromolecule. Such cooperativity reflects the interconnected nature of binding events and provides a framework for understanding non-ideal adsorption processes.			
Redlich-Peterson	$q_{e}=\frac{K_{rd1}\cdot C_{e}}{1+K_{rd2}\cdot C_{e}^{n_{rd}}}$	*q_e_*—adsorbate concentration on the solid phase at equilibrium (mg/g), *C_e_*—adsorbate concentration on the fluid phase at equilibrium (mg/L), *K_rd_*_1_—R-P constant (L/g), *K_rd_*_2_—R-P constant (L/mg), *n_rd_*—(dimensionless)		[54,56,57,58]
Model description	The Redlich-Peterson (R-P) isotherm is an empirical three-parameter hybrid model that incorporates features of both the Langmuir and the Freundlich equations. It combines a Langmuir-type linear concentration term in the numerator with an exponential Freundlich-type term in the denominator, enabling an accurate description of adsorption over a wide concentration range. Owing to this structure, the R-P model is applicable to both homogeneous and heterogeneous adsorption systems. In limiting cases, it approaches the Freundlich isotherm at high concentrations and the Langmuir model at low concentrations. Parameter estimation is typically performed using numerical optimization to best fit experimental data.			
Sips	$q_{e}=\frac{q_{s}\cdot {\left(K_{s}\cdot C_{e}\right)}^{n_{s}}}{1+{\left(K_{s}\cdot C_{e}\right)}^{n_{s}}}$	*q_e_*—adsorbate concentration on the solid phase at equilibrium (mg/g), *C_e_*—adsorbate concentration on the fluid phase at equilibrium (mg/L), *q_s_*—Sips constant (mg/g), *K_s_*—Sips constant (L/mg), *n_s_*—(dimensionless)		[54,56,57,58]
Model description	The Sips isotherm is a three-parameter hybrid model that integrates the Langmuir and the Freundlich equations to describe adsorption on heterogeneous surfaces while preventing the unrealistic, unlimited uptake predicted by the Freundlich model at high concentrations. It behaves like the Freundlich isotherm at low adsorbate concentrations and approaches Langmuir monolayer saturation at higher concentrations. The model parameters are influenced by operating conditions such as pH, temperature, and solute concentration, allowing the Sips equation to effectively represent both homogeneous and heterogeneous adsorption systems.			

Ref. *—references for each mentioned model.

**Table 5:** Determined parameters for the kinetic models assessed in this study.

Model Parameters	Initial Concentrations of MG (mg/L)				
	10	20	30	40	50
**Pseudo-first-order**					
*q_e_*	3.1113	6.1952	9.2595	12.1453	14.8091
*k*_1_	0.0551	0.0572	0.0548	0.0500	0.0581
R^2^	0.9494	0.8710	0.8964	0.9090	0.8266
**Pseudo-second-order**					
*q_e_*	3.3082	6.5876	9.8876	13.0422	15.7730
*k*_2_	0.0307	0.0159	0.0098	0.0066	0.0066
R^2^	0.9807	0.9902	0.9996	0.9994	0.9898
**Elovich**					
*α*	23.3357	46.0885	42.4030	29.4808	91.6225
*β*	3.0391	1.5200	0.9585	0.6734	0.6205
R^2^	0.8014	0.8784	0.8903	0.8967	0.9263
**Weber-Morris**					
*k*	0.0560	0.1156	0.1842	0.2630	0.2898
*B*	2.3137	4.5747	6.6759	8.4287	10.7908
R^2^	0.5989	0.7000	0.7162	0.7260	0.7733
**Pseudo-n-order**					
*q_n_*	3.6067	7.1798	10.8243	14.3685	17.2161
*k_n_*	0.0121	0.0032	0.0012	0.0006	0.0005
R^2^	0.9349	0.9730	0.9821	0.9828	0.9916
**Experimental data**					
*q_e_*	3.1552	6.3890	9.5967	12.6292	15.5677

Note: the biosorption capacity (*q_e_*) for the experimental data is expressed in mg/g.

**Table 6:** Calculated parameters of the nonlinear isotherm models for MG dye biosorption onto the SDA 5% biosorbent.

Isotherm Model Parameters			
Langmuir		Hill	
*q_l_*	30.0086	*q_hi_*	17.7054
*k_l_*	0.3628	*K_hi_*	1.1301
R^2^	0.9730	*n_hi_*	1.7860
		R^2^	0.9978
Freundlich		Redlich-Peterson	
*K_fr_*	7.7438	*K_rd_*_1_	8.4013
*n_fr_*	1.5322	*K_rd_*_2_	0.0520
R^2^	0.9437	*n_rd_*	2.2706
		R^2^	0.9890
Temkin		Sips	
*K_t_*	3.4743	*q_s_*	17.7054
*b_t_*	369.8605	*K_s_*	0.9338
R^2^	0.9968	*n_s_*	1.7860
		R^2^	0.9978

## Data Availability

The original contributions presented in the study are included in the article. Further inquiries can be directed to the corresponding authors.

## Supplementary Materials

The following supporting information can be downloaded at https://www.mdpi.com/article/10.3390/polym18091136/s1, Figure S1: UV–Vis spectra of Malachite green recorded over a wide pH range (2–12), highlighting pH-induced spectral variations and dye stability.
