# Supplementary material for: Design and Application of a Sawdust–Alginate Biocomposite for Sustainable Cationic Dyes Removal from Aqueous Solutions
Source: Polymers (Basel). 2026 May 5;18(9):1136. doi: 10.3390/polym18091136 (PMC13165466; doi:10.3390/polym18091136)
Supplement: Supplementary file 1 [file polymers-18-01136-s001.zip › polymers-4248151-supplementary.pdf]

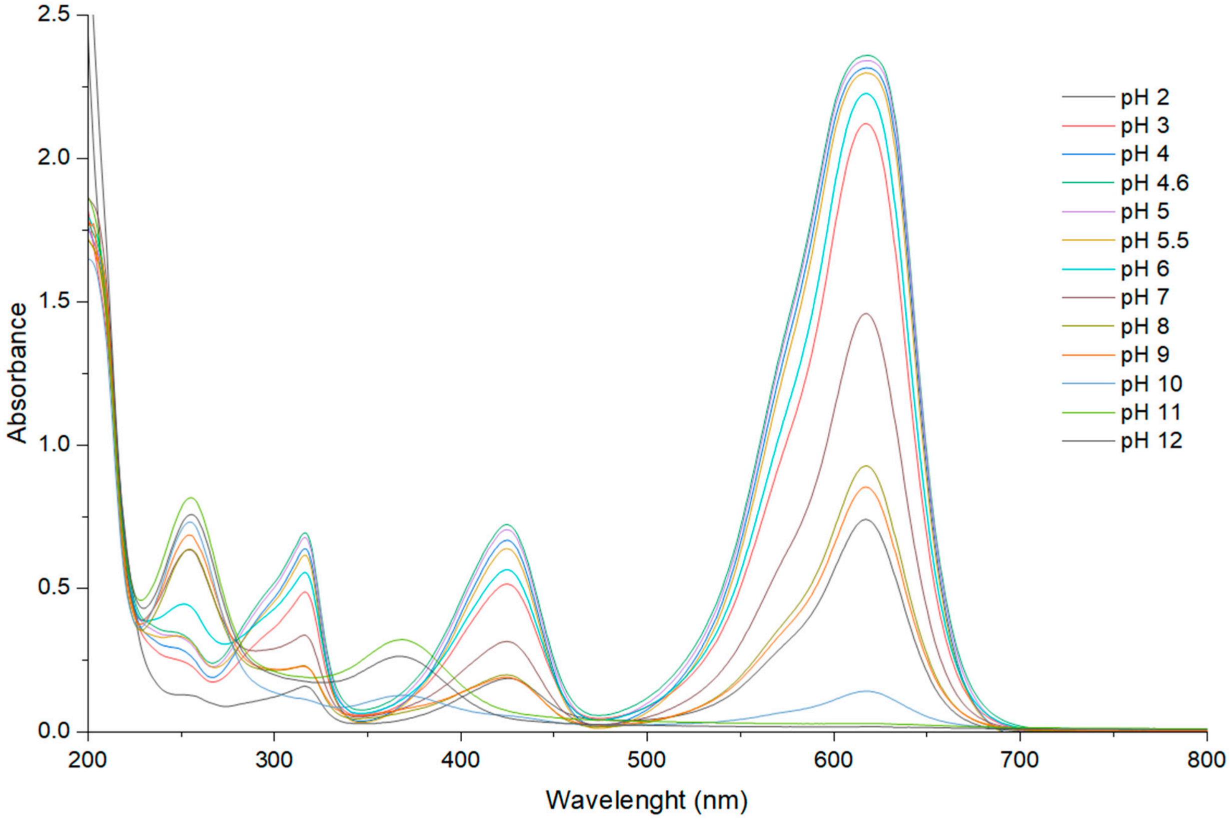

Figure S1. UV–Vis spectra of Malachite green recorded over a wide pH range (2–12), highlighting pH-induced spectral variations and dye stability.
